# Supplementary material for: Mutations in PCYT2 disrupt etherlipid biosynthesis and cause a complex hereditary spastic paraplegia
Source: Brain. 2019 Oct 22;142(11):3382–97. doi: 10.1093/brain/awz291 (PMC6821184; doi:10.1093/brain/awz291)
Supplement: awz291_Supplementary_Data [file awz291_supplementary_data.zip › awz291-Suppl_data/Supplementary_Data4.zip!/Supplementary information 3 - fibroblast lipidomics.zip!/Fibroblast lipidomics/Controle vs Patient1/Statistical plots and data/Random forest.pdf]

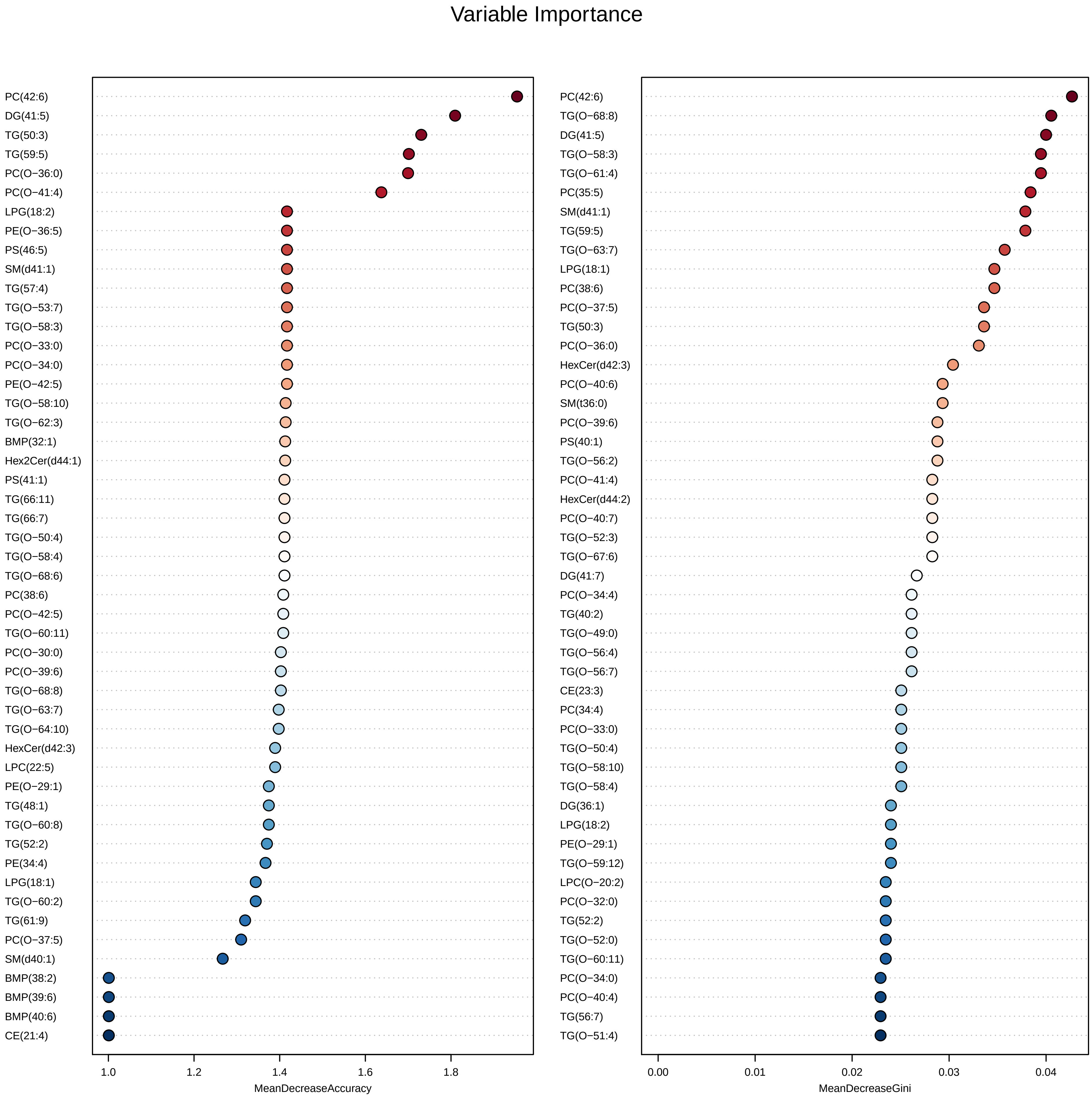
Variable Importance
PC(42:6)
DG(41:5)
TG(50:3)
TG(59:5)
PC(O−36:0)
PC(O−41:4)
LPG(18:2)
PE(O−36:5)
PS(46:5)
SM(d41:1)
TG(57:4)
TG(O−53:7)
TG(O−58:3)
PC(O−33:0)
PC(O−34:0)
PE(O−42:5)
TG(O−58:10)
TG(O−62:3)
BMP(32:1)
Hex2Cer(d44:1)
PS(41:1)
TG(66:11)
TG(66:7)
TG(O−50:4)
TG(O−58:4)
TG(O−68:6)
PC(38:6)
PC(O−42:5)
TG(O−60:11)
PC(O−30:0)
PC(O−39:6)
TG(O−68:8)
TG(O−63:7)
TG(O−64:10)
HexCer(d42:3)
LPC(22:5)
PE(O−29:1)
TG(48:1)
TG(O−60:8)
TG(52:2)
PE(34:4)
LPG(18:1)
TG(O−60:2)
TG(61:9)
PC(O−37:5)
SM(d40:1)
BMP(38:2)
BMP(39:6)
BMP(40:6)
CE(21:4)
1.0
1.2
1.4
1.6
1.8
MeanDecreaseAccuracy
PC(42:6)
TG(O−68:8)
DG(41:5)
TG(O−58:3)
TG(O−61:4)
PC(35:5)
SM(d41:1)
TG(59:5)
TG(O−63:7)
LPG(18:1)
PC(38:6)
PC(O−37:5)
TG(50:3)
PC(O−36:0)
HexCer(d42:3)
PC(O−40:6)
SM(t36:0)
PC(O−39:6)
PS(40:1)
TG(O−56:2)
PC(O−41:4)
HexCer(d44:2)
PC(O−40:7)
TG(O−52:3)
TG(O−67:6)
DG(41:7)
PC(O−34:4)
TG(40:2)
TG(O−49:0)
TG(O−56:4)
TG(O−56:7)
CE(23:3)
PC(34:4)
PC(O−33:0)
TG(O−50:4)
TG(O−58:10)
TG(O−58:4)
DG(36:1)
LPG(18:2)
PE(O−29:1)
TG(O−59:12)
LPC(O−20:2)
PC(O−32:0)
TG(52:2)
TG(O−52:0)
TG(O−60:11)
PC(O−34:0)
PC(O−40:4)
TG(56:7)
TG(O−51:4)
0.00
0.01
0.02
0.03
0.04
MeanDecreaseGini
